# Simultaneous, efficient and continuous oil–water separation via antagonistically functionalized membranes prepared by atmospheric-pressure cold plasma

**DOI:** 10.1038/s41598-021-82761-9

**Published:** 2021-02-04

**Authors:** Dong-hyun Kim, Rodolphe Mauchauffé, Jongwoon Kim, Se Youn Moon

**Affiliations:** 1 Department of Applied Plasma and Quantum Beam Engineering, Jeonbuk National University, 567 Baekje-daero, Deokjin-gu, Jeonju-si, Jeollabuk-do 54869 Republic of Korea; 2 Department of Quantum System Engineering, Jeonbuk National University, 567 Baekje-daero, Deokjin-gu, Jeonju-si, Jeollabuk-do 54869 Republic of Korea

**Keywords:** Plasma physics, Chemistry, Materials science

## Abstract

For decades, oil and water separation has remained a challenge. Not only oil spills but also industrial oily wastewaters are threatening our environment. Over the years, oil–water separation methods have been developed, however, there are still considerable hurdles to overcome to provide a low cost and efficient process able to treat a large amount of liquid. In this work, we suggest a continuous, simultaneous and effective oil–water separation method based on the antagonistic functionalization of meshes using atmospheric pressure cold plasmas. Using this robust plasma method, superhydrophobic/underwater-superoleophilic or superhydrophilic/underwater-superoleophobic functionalized meshes are obtained. Antagonistically functionalized meshes can simultaneously separate oil and water and show continuous separation flow rates of water (900 L m^−2^ h^−1^) and oil (400 L m^−2^ h^−1^) with high purities (> 99.9% v/v). This fast, low-cost and continuous plasma-based process can be readily and widely adopted for the selective functionalization of membranes at large scale for oil-spill cleanup and water purification.

## Introduction

Oil–water separation is a major challenge for oil spills cleanup and industrial oily wastewater recycling. Oil spill disasters in Taean of Korea (2007) and Gulf of Mexico (2010) show the importance of the development of efficient oil–water separation methods^1^. Current commonly used approaches to remove oil during oil spills have various drawbacks: oil burning is fast but generate smoke; the use of oil skimmers is practical however the recovered oils contain high amount of water; sorbent materials are effective but generate oil sorbent wastes to treat and dispose; the use of chemical dispersants although fast to put in application, are reported to be toxic for marine animals^2–5^. In addition to oil spills clean–up, the demand for oil–water separation methods is also increasing in other domains generating oily waste waters, e.g. food industry or factories using cutting fluids^6,7^. In response, along with the commonly used methods in these industries such as gravity separation, filtration, centrifugation, flotation or electrochemical methods, many alternative ways are being investigated in the literature^8–11^. Most of them have specific limits such as being batch processes, involving time consuming steps, being economically nonviable, producing secondary pollution due to generated by-products and so on^11^.

In order to overcome these drawbacks and provide a fast, continuous, efficient and low cost method to separate oil and water, the surface functionalized membranes are promising alternatives^11–14^. Functionalized membranes with hydrophilic/oleophobic or oleophilic/hydrophobic surfaces are permeable to water or oil, respectively, and allow to selectively separate a mixture. Since methods relying on the use of a single functionalized membrane are widely reported in literature, the systems having the membrane usually show vertically oriented configuration to rely on gravity for effective separation^11–14^. For water permeable membranes, for example, as water has a high density compared to commonly separated oils, the water is passing through the membrane while the unseparated oil remains and builds up in the system^11^. This build up shows the limits of such system for the continuous separation of oil and water. Therefore, an additional permeable membrane could be used to recover the second liquid. Through combination of both oil and water-permeable membranes, the continuous, simultaneous and selective oil and water separation is possible^15^.

The selective surface wetting properties of surfaces are commonly achieved by controlling the morphology and the surface energy through formation of a hierarchical micro-nanostructure and/or tuning of the surface chemistry^16^. To functionalize the surfaces, wet and dry methods, including dip coating, sol–gel, surface etching, electrospinning, lithography, chemical vapor deposition, plasma surface modification and radical polymerization, are reported in the literature^11,13,17–21^. Among them, atmospheric-pressure plasma-based methods are of major interest for industrial up-scaling. These methods are operating in open-air, generate few wastes, are easily implementable in continuous processes like roll-to-roll systems, are able to rapidly modify large surfaces in a single step, run at low cost as they do not require the use of expensive vacuum equipment and they have low processing temperature, allowing the modification of heat sensitive materials^22^. The highly reactive species present in the plasma enable to directly modify the surface of material or to form functional layers^13,20,22^. Via simple variation of the plasma gas composition, the surface property of meshes and membranes can easily be controlled. These methods were applied to develop oil–water separation devices relying on single functionalized membranes or meshes: Chen et al*.* reported a 97.5% oil/water separation efficiency using nylon membranes modified by a mm-size atmospheric pressure helium plasma jet to develop superhydrophilic/underwater-superoleophobic surface for water permeation^20^. You et al*.* successfully demonstrated oil collection using a stainless steel mesh functionalized via atmospheric pressure plasma polymerization to develop oleophilic/superhydrophobic surface^13^.

The plasma methods, easily up-scalable, low-cost and versatile, appear to be good candidates for the development of an advanced fabrication method for continuous and simultaneous oil–water separation. In this work, antagonistically functionalized meshes for continuous, simultaneous and selective oil–water separation were obtained by atmospheric pressure plasma deposition via simple tuning of plasma conditions. The surface of the stainless steel mesh was selectively functionalized and superhydrophobic/underwater-superoleophilic or superhydrophilic/underwater-superoleophobic properties were obtained respectively to enable oil or water to pass through. Scanning electron microscopy (SEM), water/oil contact angle (WCA/OCA) measurements, X-ray photoelectron spectroscopy (XPS) and Fourier transform infra-red (FTIR) analysis were performed to investigate the morphology and surface chemistry of the modified meshes. The separation performance in an oil–water separation device was assessed by investigating the separation flow rate stability and the purity of separated liquids over an extended period of time.

## Results and discussion

### Surface functionalization by atmospheric-pressure plasma

Stainless steel meshes were functionalized by atmospheric pressure plasma deposition with case 1 and case 2 as described in Table 1. To form functional surfaces, hexamethyldisilazane (HMDS 99.9%, Sigma-Aldrich) and titanium isopropoxide (TTIP 99.9%, Sigma-Aldrich) precursors were introduced in the plasma using carrier gases.

**Table 1:** Atmospheric-pressure plasma deposition parameters.

Condition	RF power (W)	Passes number	Gas flow rate (L min^−1^)		
			Dilution gas	Carrier gas	Additional gas
Case 1	200	50	He	He carrying HMDS vapors	N_2_
			20	0.034	0.6
Case 2	40	60	Ar	Ar carrying TTIP vapors	O_2_
			9	1	0.005

**Figure 1:**
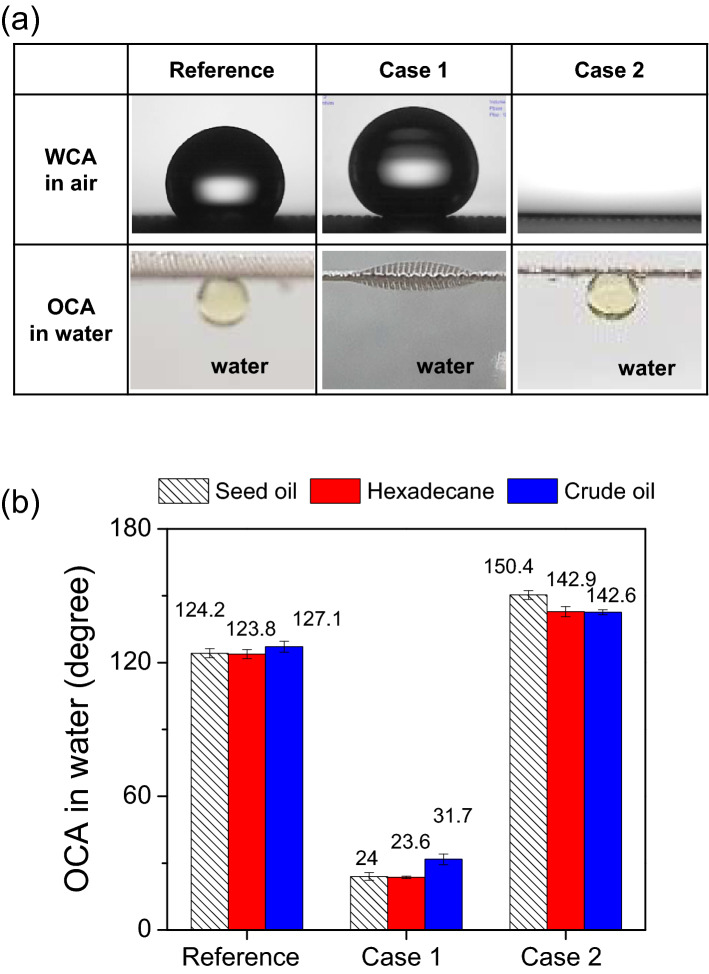
(**a**) Water contact angle in air and oil contact angle in water for untreated mesh (reference), plasma treated mesh with case 1 and case 2. (**b**) Oil contact angle in water on the meshes were also measured with various oils such as seed oil, hexadecane and crude oil.

Figure 1a shows photographs of water and seed oil droplets on a reference mesh (untreated) and meshes plasma treated with case 1 and case 2. While the WCA in air and OCA in water of a reference mesh, were 130.5° and 124.2°, respectively, upon plasma treatment with case 1, the surface exhibited a superhydrophobic (161.0° WCA) and underwater-superoleophilic behavior (24° OCA in water) as seen in Fig. 1a.

**Figure 2:**
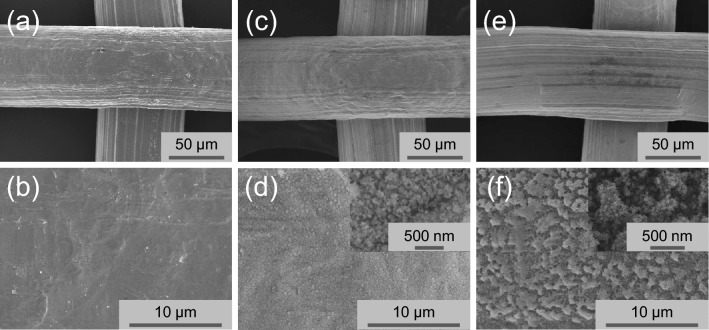
SEM images of an untreated stainless steel mesh (**a**, **b**), plasma treated mesh with case 1 (**c**, **d**) and case 2 (**e**, **f**).

Even though both meshes showed superhydrophobic behavior at first when measurements are performed on dried meshes, the reference mesh easily lost its hydrophobicity by water dipping, and showed almost 0° WCA by cyclic dipping in water (Supplementary Fig. S1). For the reference mesh, these hydrophobic properties observed for a dried surface are likely to come from the presence of a slight surface roughness (cf. SEM observations) together with some hydrophobic compounds adsorbed on the surface coming from atmospheric contamination as reported in literature^23^. Upon immersion in the water these weakly bonded compounds do not play any more role and the hydrophilic surface and its roughness leads to underwater-oleophobicity^23^. On the other hand, the plasma treated mesh with case 1 retained its superhydrophobic behavior in cyclic water dipping test (Supplementary Fig. S1), highlighting the importance of surface modification to retain the desired surface properties. By contrast, when the mesh was functionalized using case 2 plasma condition, water droplet spread completely on the treated mesh in air and oil droplet exhibited a 150.4° OCA in water. Although all meshes, untreated or treated by case 1 and case 2, show oleophilic surface properties in air, the meshes exhibited different OCA values in water (Fig. 1a). OCA in water for various oils such as seed oil, hexadecane and crude oil were similar, respectively, on meshes (Fig. 1b) modified with case 1 and case 2, which means the selective and continuous oil–water separation could be realized simultaneously. Dunderdale et al*.* reported these antagonistic behaviors are promising for an effective continuous oil–water separation^15^. Oil is only able to permeate case 1-modified meshes with superhydrophobic/underwater-superoleophilic surface while water is only able to permeate case 2-modified mesh with superhydrophilic/underwater-superoleophobic surface. It is well known that the surface wettability for water and/or oil is based on the synergetic effects of physical morphology and surface chemistry. Thus, to investigate the morphology and surface chemistry of the modified meshes by plasmas, SEM, FTIR, and XPS measurements were conducted. Figure 2 shows the SEM images of the reference mesh and treated meshes with different magnifications. The untreated reference mesh (Fig. 2a,b) looks relatively smooth but at the micrometer scale (Fig. 2b), only some bumps and trenches originating from manufacturing can be observed. At low magnification, the mesh surfaces modified by case 1 (Fig. 2c) or case 2 (Fig. 2e) look similar to the surface of the reference mesh, however for higher magnifications (Fig. 2d,f), the surface morphology of the stainless steel meshes dramatically changes. For case 1 (Fig. 2d and inserted image), a layer of uniformly distributed nano-aggregates with the size of several tens of nanometers is present on the surface of the mesh wires and for case 2, micro-nano hierarchical structures consisting of microscale bumps formed by nanoscale particles are observed. These structures are likely to be formed due to reactions of the precursor in the gas phase rather than on the surface, thus leading to the formation and aggregation of particles before deposition on the surface^24^. These types of morphologies are known to play an important role in inducing superhydrophobicity or superoleophobicity in combination with surface chemistry and are thus important for oil–water separation^13,16,25,26^.

The surface chemistry is an important parameter for surface wetting control^15^. Hence, the surface chemistry of the deposited layers was investigated via XPS and FTIR analyses. To do so, layers were deposited on silicon wafers. The chemical composition of the layers resulting from the dissociation of HMDS (case 1) and TTIP (case 2) in the plasma were assessed by XPS analyses, after surface cleaning by argon sputtering to remove surface adventitious carbon, and are summarized in Table 2. For case 1, while Si and O are largely present in the layer, a high amount of carbon is also observed.

**Table 2:** Elemental compositions of thin films deposited with case 1 and 2 conditions.

Case 1		Case 2	
Element	Composition (at.%)	Element	Composition (at.%)
Si	34	Ti	25
O	33	O	60
C	26	C	14
N	7	N	1

**Figure 3:**
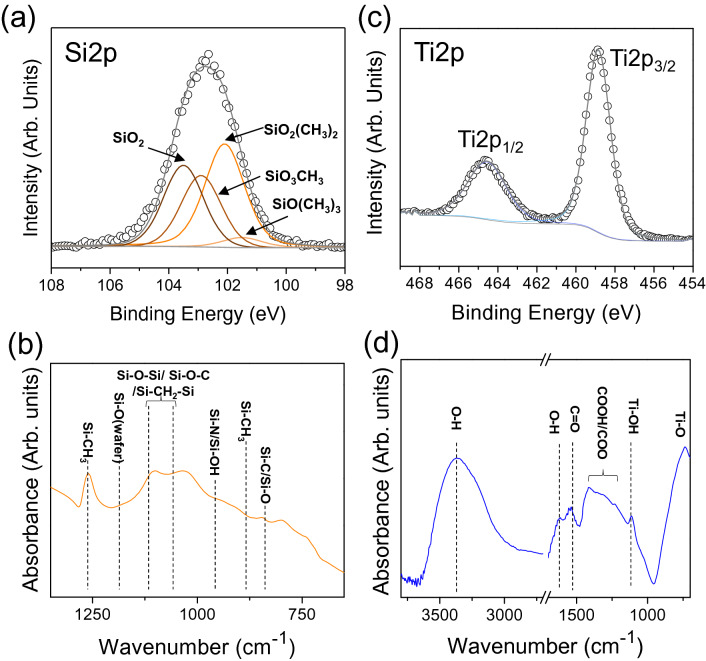
XPS fitting of (**a**) Si2*p* and (**c**) Ti2*p* core levels for thin films deposited with case 1 and case 2 respectively. FTIR spectra of thin films deposited using (**b**) case 1 and (**d**) case 2 conditions.

To get insight into the chemistry of the layer, the Si2*p* core level (before Ar sputtering, to avoid changes in the bonds environment) representing the silicon environment in the layer, is fitted as seen in Fig. 3a. For the sake of clarity, Si2*p*_3/2_ and Si2*p*_1/2_ peaks due to spin–orbit splitting for each state are gathered in a single contribution. While a contribution at 103.5 eV corresponding to silicon in a Si^4+^ state as in the Si–O–Si network of SiO_2_, is present, the presence of lower oxidation states contributions at 102.9 eV, 102.1 eV, 101.5 eV could be attributed, respectively, to the presence of methyl groups bonded to silicon as in SiO_3_CH_3_, SiO_2_(CH_3_)_2_, SiO(CH_3_)_3_^27^. These data are supported by FTIR analyses (Fig. 3b), where the presence of peaks in the 1035 cm^−1^–1100 cm^−1^ region corresponding to the formation of a Si–O–Si/Si–O–C network are present as well as a sharp peak at 1260 cm^−1^ assigned to Si-CH_3_ vibrations^27,28^. These methyl groups are likely to mainly come from the incomplete dissociation of HMDS where Si–CH_3_ is present^28,29^. On the other hand, for case 2, Ti and O are the main components with about 14% carbon present in the layer. The Ti2*p* XPS core level fitting (Fig. 3c) shows two components at 458.9 eV (Ti2*p*_3/2_) and 464.6 eV (Ti2*p*_1/2_). The peak separation value between Ti2*p*_3/2_ and Ti2*p*_1/2_ is 5.7 eV suggesting the presence of Ti under Ti^4+^ state, confirming the existence of TiO_2_ on the sample. FTIR analysis (Fig. 3d) confirms the presence of Ti–O bonds (539 cm^−1^) in the deposited layers as well as hydrophilic groups such as OH, observed at 1620 cm^−1^ and 3350 cm^−1^ and at 1110 cm^−1^ in Ti–OH bonds^30^. Based on the morphology observations and surface chemical analyses, the presence of non-polar groups (CH_3_), in case 1 and polar groups (OH) in case 2, combined with their specific surface morphologies observed by SEM (Fig. 2), seems to be responsible for the different surface wettability.

### Separation performance of functionalized meshes

**Figure 4:**
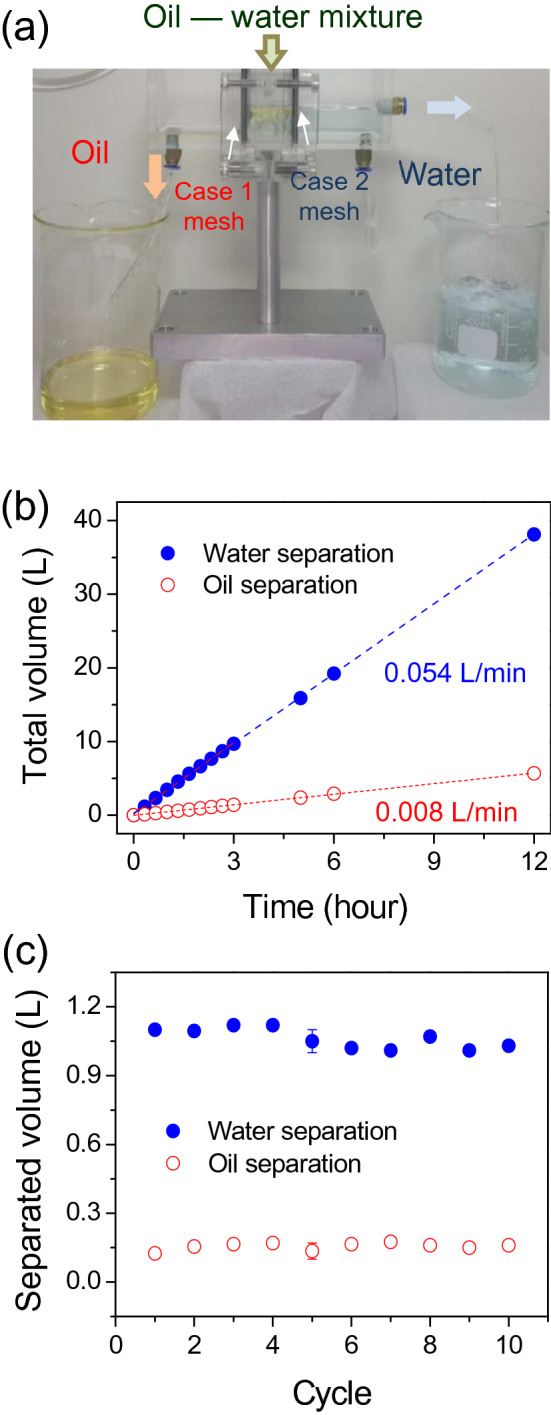
(**a**) Picture of the device during continuous and simultaneous oil–water separation experiment. Water was dyed with methylene blue. (**b**) Measurements of accumulated volumes of separated oil and water during a 12-h separation process. (**c**) Cyclic test of separating stability by collecting the separated liquid for 20 min repetitively.

To demonstrate the performance of functionalized meshes for efficient oil–water separation, the intrusion pressure, maximal permeation rates of each liquid, flow stability and purity of filtrated oil and water were studied. First, the performance of each mesh was individually assessed. The maximal permeation flow rate of oil or water through each functionalized mesh was measured as described in the experimental section. Maximal permeation flow rate of 3830 L m^−2^ h^−1^ for oil collection (case 1) and 400,000 L  m^−2^ h^−1^ for water collection (case 2) were measured. In this experiment, the same mesh size (average hole size 124 μm) is used for case 1 and 2, the coatings thickness being in the order of one micrometer for both cases, only a negligible reduction of the mesh hole size is likely to occur. The lower flow rate for oil is then likely to be due to its higher viscosity. Indeed, soybean oil is reported to have a viscosity of about 54 cP at 23.9 °C while water has a 0.89 cP viscosity at 25 °C^31,32^. High flow rate is interesting to have a fast separation device however another parameter is important to sustain a stable selective separation: the intrusion pressure. This parameter shows the capacity of a membrane to avoid another liquid other than the targeted liquid to permeate through the membrane. The intrusion pressures for water in case 1 and oil in case 2 were measured with a homemade apparatus (Supplementary Fig. S2) as described in the experimental section. For a dried reference mesh, i.e. untreated, the intrusion pressure for water was 460 Pa, however a water-wet reference mesh led to almost direct penetration of the water, thus about 0 Pa. This result is in accordance with the loss of hydrophobicity observed previously upon cyclic water dipping test (Supplementary Fig. S1). It is worth noting that intrusion pressure value is also related to the mesh size and can be compared only in the case of similar mesh sizes. As plasma deposition leads to deposition of really thin layers on the mesh, in the order of the micrometer, a negligible reduction of the hole size is occurring upon treatment and any significant variation of intrusion pressure is likely to be due to the effect of the deposited film on the surface. For case 1 modified meshes with superhydrophobic properties, a water intrusion pressure of 1400 Pa was obtained. For case 2 treated meshes, a 2700 Pa underwater oil intrusion pressure was measured, more than 8 times higher than reference meshes oil intrusion pressure (320 Pa) also showing underwater-superoleophobicity. This difference is likely to come from the high surface roughness of case 2 modified meshes, able to retain a thin layer of water conferring stable superoleophobicity in water, compared to the smoother reference meshes, where the thin water layer is less stable and the mesh become more easily wetted by oil under the oil pressure^15,24^. The purity of separated liquids by each membrane was assessed in individual experiments. As depicted in experimental section, the stability of the separation process as well as the purities of the separated liquids in a continuous separation setup were investigated (Fig. 4a). A mixture of oil and water was poured into the mixing bath (center) with fixed flow rates for water (0.055 L min^−1^) and oil (0.008 L min^−1^). As seen in Supplementary Movie 1 and in Fig. 4a, water dyed with methylene blue on the right and oil on the left, are successfully continuously separated for 12 h. As shown in Fig. 4b, the separated water or oil volumes were linearly increased during the separation process. The separating flow rates of water and oil close to the input flow rates values, i.e. 0.054 L min^−1^ for water and 0.008 L min^−1^ for oil as determined by the slopes of the data. These output flow rates, linear with respect to time and close to the values of the input flow rates suggest that no accumulation of liquid is occurring in the center mixing bath and also suggest a good purity of the separated liquids indirectly. Figure 4c depicts the volumes of the collected oil and water during 10 repetitions of oil–water separation experiments. For each test, the separated oil and water was collected for 20 min to get the measurable volume of liquid. In the cyclic test, the separated water volume was slightly decreased and then seems to reach a plateau, likely to be due to the aging behavior of the plasma modified superhydrophilic surface. However, the average volume of separated water and oil collected for 20 min was 1062 mL and 156 mL respectively, which was similar to the controlled input flow rates of water (0.055 L min^−1^) and oil (0.008 L min^−1^).

**Figure 5:**
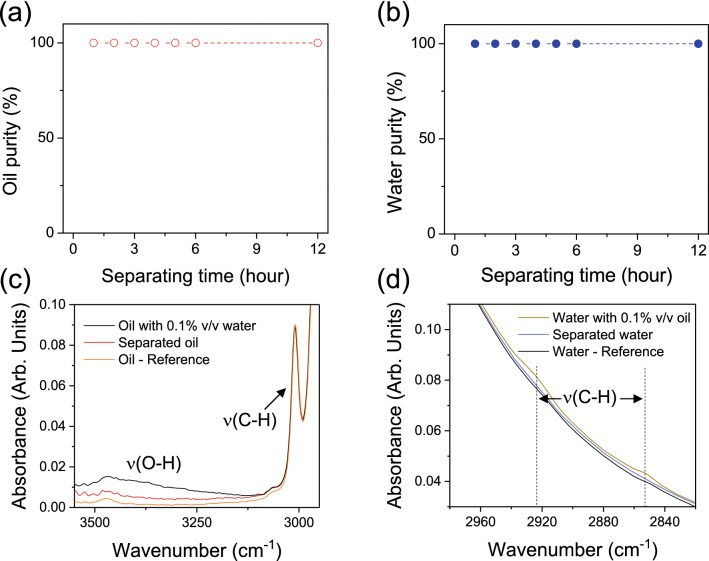
Purity of separated (**a**) oil and (**b**) water determined by distillation of liquids collected after separation time ranging from 0 to 12 h. (**c**) FTIR spectra comparison between separated oil (red line) and a 0.1%v/v water in oil reference solution (black line). (**d**) FTIR spectra comparison between separated water (blue line) and a 0.1%v/v oil in water reference solution (dark yellow line).

The purities of the separated liquids in a continuous separation setup were investigated through distillation at first. Small quantities of liquids were regularly collected at the output of the device during the separation process for 12 h. Distillation of the collected liquids was performed to estimate the purity as depicted in experimental. The purity both for oil (Fig. 5a) or water (Fig. 5b) remained above 99.9% during the whole measurement period showing the high stability of the meshes for separation. Since the degree of purity via distillation is limited by the resolution of the scale used in this work, FTIR analysis was also investigated to further elucidate the purity of separated liquids. In full spectral ranges, FTIR spectra of separated oil and water seem to perfectly coincide with the spectra of pure oil and water, respectively (Supplementary Fig. S3). The FTIR spectra of separated liquids collected after 3 h of separation are also compared to reference solutions, *i.e.* oil with 0.1% v/v water (in case 1) or water with 0.1% v/v oil (in case 2) as shown in Fig. 5c and d. On Fig. 5c, while the reference solution, i.e. oil with 0.1% v/v water, displays a high absorbance in the OH region (around 3350 cm^−1^), we can see that for separated oil the absorbance in the OH groups region is drastically reduced, with absorbance close to pure oil. This suggests that the separated oil has a very low water content. Similarly, on Fig. 5d, peaks at 2852 and 2925 cm^−1^ corresponding to the presence of CH bonds from the oil are observable in the case of the 0.1% v/v of oil in water while for the separated water they are less noticeable, very similar to the reference spectrum of pure water. It is then reasonable, according to these results, to estimate that the liquids separated by our device have purities above 99.9% v/v. The surface modification by atmospheric-pressure cold plasma is reported to be an efficient way to simply modify the surface of meshes in open air to provide antagonistic surface properties for continuous and simultaneous selective oil–water separation with high purity. From similar results on the plasma treated meshes for crude oil as shown in Fig. 1b, it could be expected the crude oil is also successfully separated from the mixture through the membranes (Supplementary Movie 2). The technical simplicity, up scalability, environmental friendly aspect of plasma assisted deposition offer a practical solution for the simple formation of functionalized meshes with promising performances for pollution control or reuse of oil or water in industries.

## Conclusions

Antagonistic mesh membranes functionalized by low-temperature atmospheric-pressure plasmas were developed for continuous, simultaneous, and selective oil–water separation. Through the simple tuning of the plasma chemistry, a 10-min plasma treatment in open air enabled the fabrication of oil–water separating membranes having superhydrophobic/underwater-superoleophilic or superhydrophilic/underwater-superoleophobic properties, respectively. The physical and chemical analyses of the membranes clearly highlighted the importance of the synergetic effects between surface chemistry and morphology to obtain different surfaces’ wettability for water and oil. The plasma modified meshes continuously separated oil and water over 12 h with the high separation purities (> 99.9% v/v) and also performed the maximal permeation flow rates of water (400,000 L m^−2^ h^−1^) and oil (3830 L m^−2^ h^−1^) which promises the modified meshes neglect the reduction of the hole size by the deposited thin film. This fast, low-cost and continuous plasma-based process is easily up-scalable and can be readily and widely adopted for the mass production of selective functionalized membranes for oil-spill pollution control or water purification in industries.

## Methods

### Materials

Stainless steel meshes (mesh number 120, average pore size 124.0 ± 0.5 μm) were cleaned with deionized water (DI) and isopropyl alcohol (IPA) and dried under compressed air prior to functionalization by plasma treatment. For plasma generation at atmospheric pressure, Ar (99.999%), He (99.999%), N_2_ (99.95%), O_2_ (99.995%) were used. Hexamethyldisilazane (HMDS 99.9%, Sigma-Aldrich) and titanium isopropoxide (TTIP 99.9%, Sigma-Aldrich) precursors were used as received without further purification. To avoid TTIP condensation in the delivery system, the temperatures of TTIP bubbler and gas pipes were heated at 70 °C and 80 °C, respectively. Seed oil (Dongwon Home Food), hexadecane (Alfa Aesar) and crude oil (SK Lubricants Co. Ltd) were used to evaluate the oil contact angle and for oil–water separation experiments.

### Atmospheric-pressure plasma functionalization

**Figure 6:**
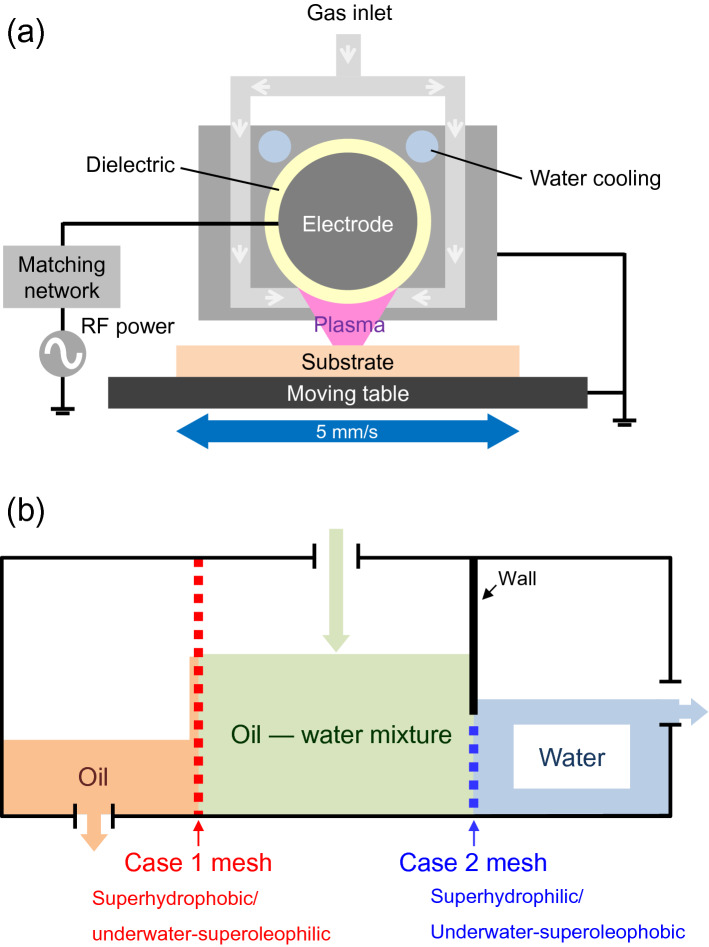
Schematic illustration of (**a**) the cross-sectional view of the atmospheric-pressure plasma source and (**b**) configuration of the oil–water separation system.

The surface of meshes were functionalized using atmospheric pressure plasma sources consisting of a high voltage cylindrical power electrode (12 mm diameter and 100 mm length) covered by a dielectric tube (2 mm thickness) as illustrated in Fig. 6a. To avoid cross contamination due to the use of different precursors, two different sources with similar geometry were used. The plasmas were generated by a 13.56 MHz radio-frequency (RF) generator (Cesar RF generator, Advanced Energy) with an impedance matching network to maintain the reflected power under 1%. The gap distance between the power electrode and the substrate placed on the bottom moving plate is set to 1 mm. Both the source body and the moving plate are electrically grounded. The substrate, i.e. mesh, was fixed on the moving plate and the deposition was performed dynamically by passing back and forth under the plasma at a 5 mm s^−1^ speed. The deposition conditions are reported in Table 1. Meshes were modified using 2 conditions, namely case 1 and case 2, using respectively He/HMDS/N_2_ and Ar/TTIP/O_2_ plasmas. The modification are performed for 50 and 60 passes under the discharges for both cases respectively.

### Characterization

Water and oil contact angles, respectively called WCA and OCA, were measured at room temperature using a contact angle measuring instrument (SmartDrop, Femtofab Ltd., Korea). Deionized water and various oils were respectively used for WCA and OCA measurements. Water contact angles in air were determined from images using the SmartDrop software and OCA in water were determined using ImageJ (NIST). The mesh surface morphology was observed by a field emission scanning electron microscope (FE-SEM, Hitachi SU-8030). The elemental compositions of the deposited films were investigated by X-ray photoelectron spectroscopy (XPS, Thermo Fisher Scientific K-Alpha). The C–C/CH peak in the C1s spectra are calibrated at 284.8 eV. More details about the chemistry of the films were studied via Fourier transform infrared spectroscopy (FTIR, Thermo Fisher Scientific Inc., Nicolet iS 50) using Attenuated Total Reflectance (ATR) mode.

### Oil and water intrusion pressure measurement

The intrusion pressures of the mesh membranes in the case of water and oil were measured using the device described in Supplementary Fig. S2. For water intrusion pressure measurements, water was inserted slowly with 1 mL at a time from the top of the tube until the water started passing through the membrane. The intrusion pressure *P*_*intrusion*_ in Pascal of the water was calculated using the Eq. (1):

1 $$P_{intrusion} = \frac{{\rho_{w} V_{w} g}}{S}$$

where ρ_*w*_ is the density of the water in kg m^−3^, *V*_*w*_ is the volume of the water in m^3^ introduced to lead to membrane intrusion, *g* is the acceleration of gravity (9.8 m s^−2^), and *S* is the area of the mesh in m^2^. In the case of oil intrusion pressure, the device was immersed in a water bath. The oil is introduced at the bottom of the device with 1 mL at a time until the critical volume leading to mesh intrusion is reached. Therefore, the oil intrusion pressure was measured by calculating the buoyant force of the oil in the water and dividing by the mesh surface as represented in Eq. (2):

2 $${P}_{oilintrusion}=\frac{{F}_{b}}{S}=\frac{{\rho }_{o}{V}_{o}g}{S}$$

where *F*_*b*_ is the buoyant force of the oil in water, ρ_*o*_ s the density of the oil, *V*_*o*_ is the volume of the oil when intrusion occurs and *S* is the area of the mesh.

### Separation efficiency assessment of meshes

Separation efficiency of functionalized mesh membrane was investigated in a lab-made separation system as illustrated in Fig. 6b. Both flow rate and purity of the separated oil or water were investigated. Meshes treated using case 1 and case 2 conditions respectively were vertically inserted to divide the system into an oil collecting bath (left), a mixing bath (center), and a water collecting bath (right). The size of the inserted meshes were 6 cm × 6 cm for case 1 and 6 cm × 2 cm for case 2. The later one has smaller height in order to ensure that the mesh is always wet in the lab-made separation system. Therefore, the water collecting bath (right) was partially filled with water to wet the mesh membrane before separation process.

The maximum separation flow rate (L m^−2^ h^−1^) capacity of each membrane was measured by injecting either water or oil and tuning the input liquid flow rate to reach a steady state, i.e. where the height of the liquid located in the mixing bath does not vary. The output flow rate is then measured and the maximal permeation rate of each liquid is obtained.

The purity of the continuously and simultaneously separated oil and water were at assessed using distillation method. Oil and water were introduced together into the mixing bath (center) with fixed input flow rates (water = 55 mL min^−1^, oil = 8 m L min^−1^). The separated liquids were collected in small glass bottles after 1, 2, 3, 4, 5, 6 and 12 h of separation and their purities were analyzed through sample distillation. By heating the bottles above the boiling point of water (100 °C) at 150 °C with a hot plate (Corning, PC-420D) during 24 h, the water present in the bottles is evaporated while the seed oil, with a smoking point above 200 °C remains unaltered. The weight of the empty bottles and the weight before and after distillation were measured using an electronic scale (OHAUS EX224G, 0.1 mg resolution) and purities were calculated using the following equations:

3 $$Oil\;purity\left( {\% w/w} \right) = \frac{{m_{distilled\;liquid} }}{{m_{sampled\;liquid} }} \times 100 = \frac{{m_{bd} - m_{b} }}{{m_{bsl} - m_{b} }} \times 100,$$

4 $$Water\;purity\left( {\% w/w} \right) = \left( {1 - \frac{{m_{distilled\;liquid} }}{{m_{sampled\;liquid} }}} \right) \times 100 = \left( {1 - \frac{{m_{bd} - m_{b} }}{{m_{bsl} - m_{b} }}} \right) \times 100$$

where *m*_*b*_ is the weight of the empty bottle, *m*_*bd*_ is the weight of the bottle containing the liquid after distillation and *m*_*bsl*_ is the weight of the bottle containing the liquid sampled from the separation device.

The purity of separated liquids was also quantitatively verified with FTIR analysis. To investigate the detection limit of FTIR analysis for purity evaluation, the spectra of collected liquids are also compared to pure liquids and reference solutions, i.e. a 0.1% (v/v) water in oil solution and a 0.1% (v/v) oil in water solution, respectively.

## Supplementary Information

Supplementary Video 1.

Supplementary Video 2.

Supplementary Information 1.
