# Publisher Correction: Simultaneous, efficient and continuous oil–water separation via antagonistically functionalized membranes prepared by atmospheric‑pressure cold plasma

**DOI:** 10.1038/s41598-021-89591-9

**Published:** 2021-05-03

**Authors:** Dong‑hyun Kim, Rodolphe Mauchauffé, Jongwoon Kim, Se Youn Moon

**Affiliations:** 1 Department of Applied Plasma and Quantum Beam Engineering, Jeonbuk National University, 567 Baekje‑daero, Deokjin‑gu, Jeonju‑si, Jeollabuk‑do 54869 Republic of Korea; 2 Department of Quantum System Engineering, Jeonbuk National University, 567 Baekje‑daero, Deokjin‑gu, Jeonju‑si, Jeollabuk‑do 54869 Republic of Korea

Correction to: *Scientific Reports* 10.1038/s41598-021-82761-9, published online 04 February 2021

The original version of this Article contained an error in Figure 2 where the scale bar labels were omitted. The original Figure 2 and accompanying legend appear below.

**Figure 2:**
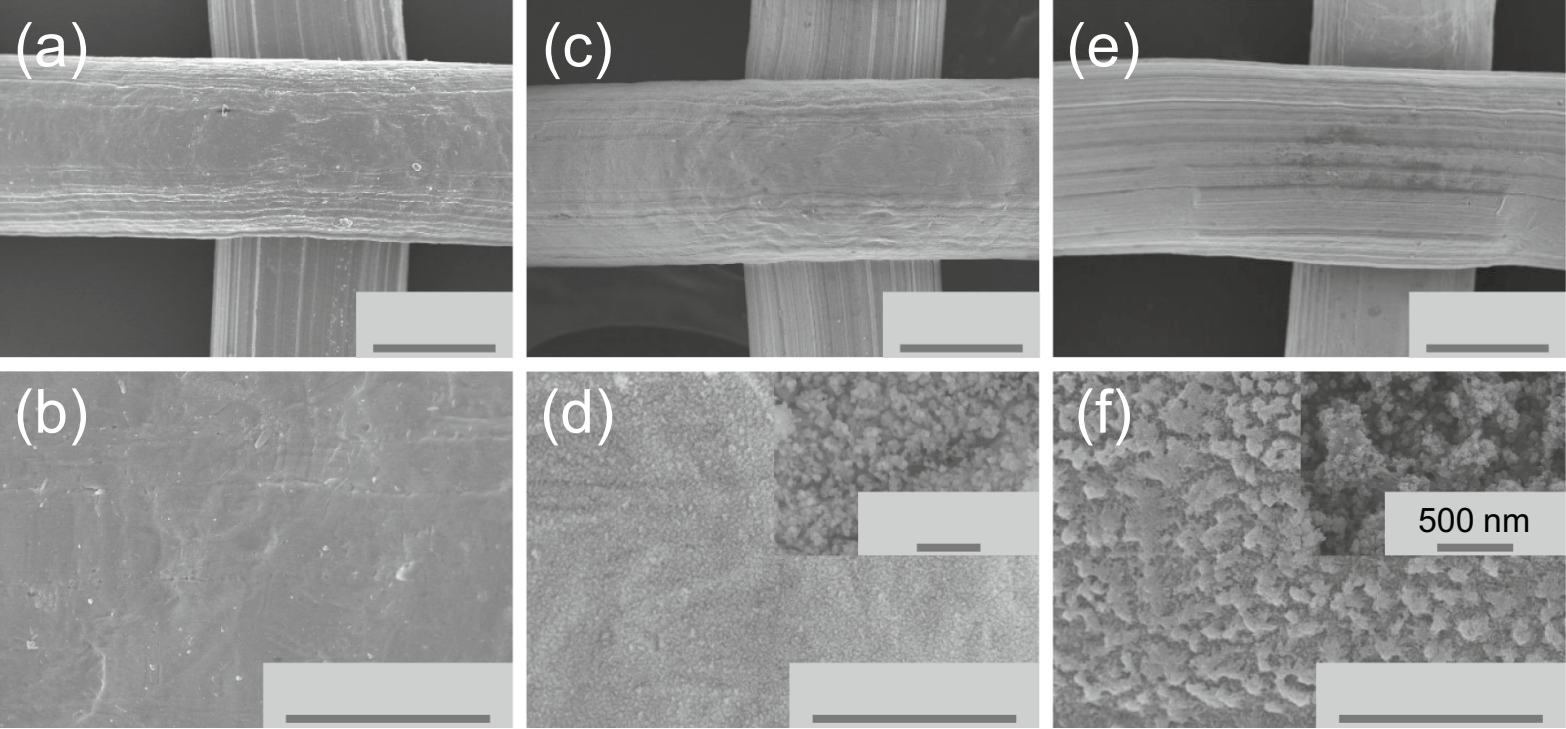
SEM images of an untreated stainless steel mesh (**a**, **b**), plasma treated mesh with case 1 (**c**, **d**) and case 2 (**e**, **f**).

The original Article has been corrected.

