# Supplementary material for: Simultaneous, efficient and continuous oil–water separation via antagonistically functionalized membranes prepared by atmospheric-pressure cold plasma
Source: Sci Rep. 2021 Feb 4;11:3169. doi: 10.1038/s41598-021-82761-9 (PMC7862374; doi:10.1038/s41598-021-82761-9)
Supplement: Supplementary file 3 — Supplementary Information 1. [file 41598_2021_82761_MOESM3_ESM.pdf]

SUPPORTING INFORMATION

Simultaneous, efficient and continuous oil-water separation via antagonistically functionalized membranes prepared by atmospheric-pressure cold plasma

*Dong-hyun Kim[1], Rodolphe Mauchauffé[2], Jongwoon Kim[1], Se Youn Moon[1,2,]**

[1]Department of Applied Plasma and Quantum Beam Engineering, Jeonbuk National University, 567 Baekje-daero, Deokjin-gu, Jeonju-si, Jeollabuk-do, Republic of Korea

[2]Department of Quantum System Engineering, Jeonbuk National University, 567 Baekje-daero, Deokjin-gu, Jeonju-si, Jeollabuk-do, Republic of Korea

*Corresponding Author: symoon@jbnu.ac.kr

Figure S1. (Left) Water contact angle on the dried untreated (reference) mesh and plasma treated mesh with case 1. (Right) Water contact angle on the reference and the plasma treated mesh after water dipping cycle.

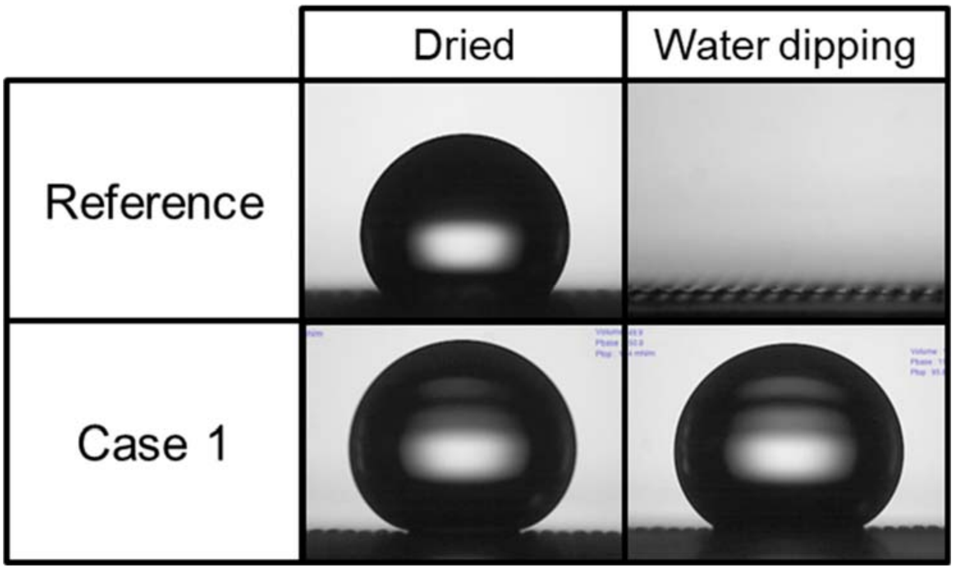

Figure S2. **S**chematic illustration of the homemade equipment to measure the intrusion pressure of plasma functionalized meshes for **(**a) water and (b) oil.

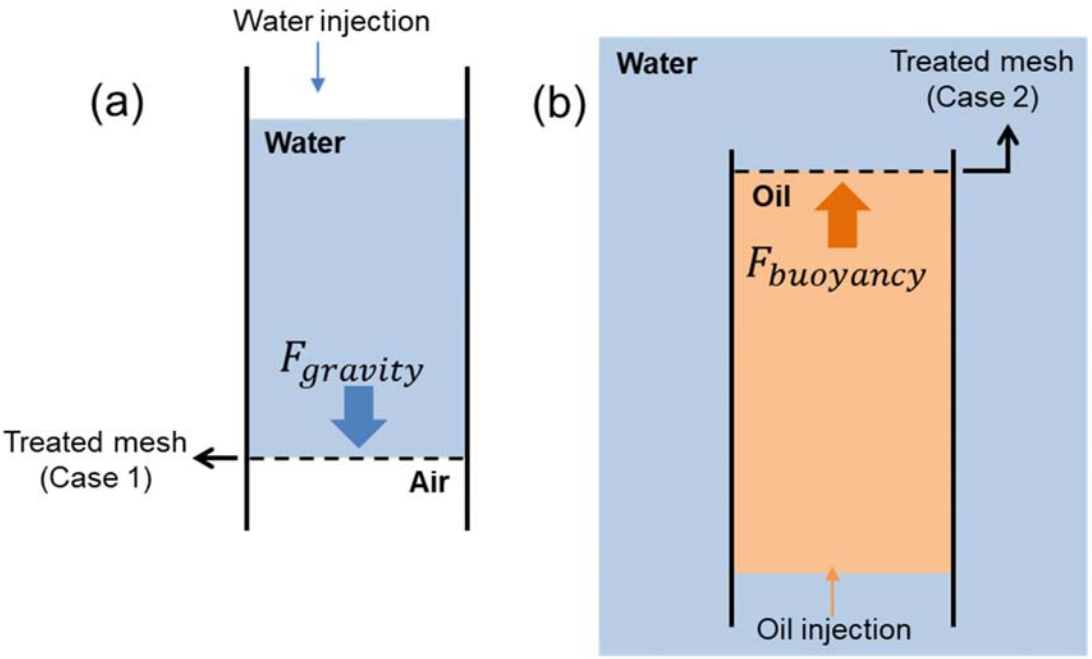

Figure S3. (a) FTIR analysis of pure water (top) and separated water (bottom) and (b) FTIR analysis of pure oil (top) and separated oil (bottom).

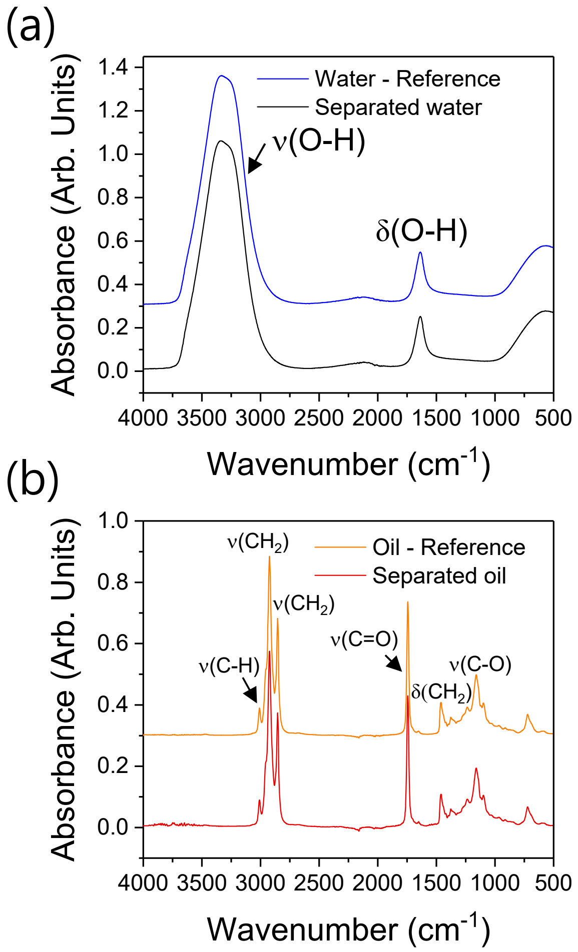

Supplementary Movie1. Continuous and simultaneous selective seed oil-water separation performed with the meshes functionalized by atmospheric-pressure plasmas with case 1 and case 2.

Supplementary Movie2. Selective separation of crude oil and water performed with the meshes functionalized by atmospheric-pressure plasmas with case 1 and case 2.
